# Discontinuation of aspirin before non-cardiac surgery causing ventricular mural thrombus for a patient with left ventricular aneurysm: a case report

**DOI:** 10.1186/s12872-020-01643-6

**Published:** 2020-08-14

**Authors:** Linger Huang, Kaili Wang, Shaojun Zhu, Kuirong Wang, Yanfeng Zhou

**Affiliations:** 1 Department of Anesthesia, First Affiliated Hospital, Zhejiang University School of Medicine, 79 Qingchun Road, Hangzhou, 310003 China; 2 Department of Ultrasound, First Affiliated Hospital, Zhejiang University School of Medicine, 79 Qingchun Road, Hangzhou, 310003 China

**Keywords:** Left ventricular aneurysm, Mural thrombus, Aspirin, Echocardiography, Non-cardiac surgery

## Abstract

**Background:**

Left ventricular mural thrombus (LVMT) is a life-threatening complication in patients with left ventricular dysfunction.

**Case presentation:**

A 67-year-old man had a history of penetrating myocardial infarction and left ventricular aneurysm (LVA). The patient was scheduled for a non-cardiac surgery and stopped aspirin for 10 days to reduce the risk of bleeding. Fresh LVMT was revealed via the transesophageal echocardiography (TEE) after the preoperative discontinuation of aspirin.

**Conclusions:**

Perioperative repeated evaluation for the thrombosis by echocardiography is essential in cases of patients with cardiovascular disease undergoing non-cardiac surgery. In high risk patient, during temporary interruption of antiplatelets, bridging with perioperative low-molecular-weight heparin is advisable.

## Background

Over the past decade despite great improvements in prognosis, myocardial infarction remains high rates of morbidity and mortality worldwide. Left ventricular aneurysm (LVA) is a serious complication of transmural myocardial infarction, resulting in lower cardiopulmonary efficacy and poorer quality of life. These patients usually having cardiac stents are strictly advised to continue antiplatelet therapy such as aspirin to prevent any thrombotic events and allow re-endothelialization [1, 2].

However, continuation or stop aspirin before elective surgery has long been in dispute. Perioperative withdraw antiplatelet therapy increases the incidence rate of thromboembolic in patients with left ventricular aneurysm. Continued use of aspirin therapy results in a higher risk of surgical bleeding [3]. In this case report, we describe a 67-year-old man diagnosed of LVA with mural thrombus via transesophageal echocardiography (TEE) due to stop aspirin for 10 days before rectal surgery.

## Case presentation

**Fig. 1:**
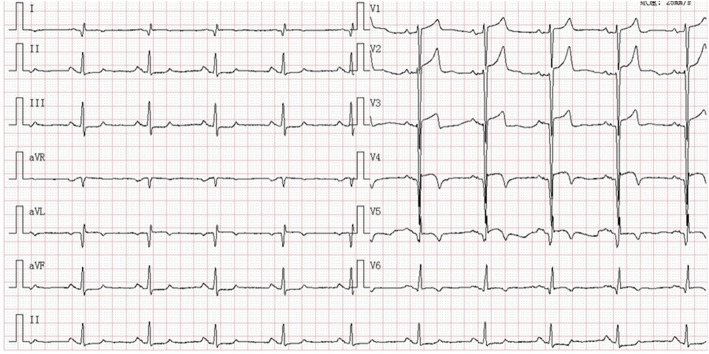
Electrocardiogram showing pathological Q waves and ST elevation with relevant inverted T waves in the anterior leads

**Fig. 2:**
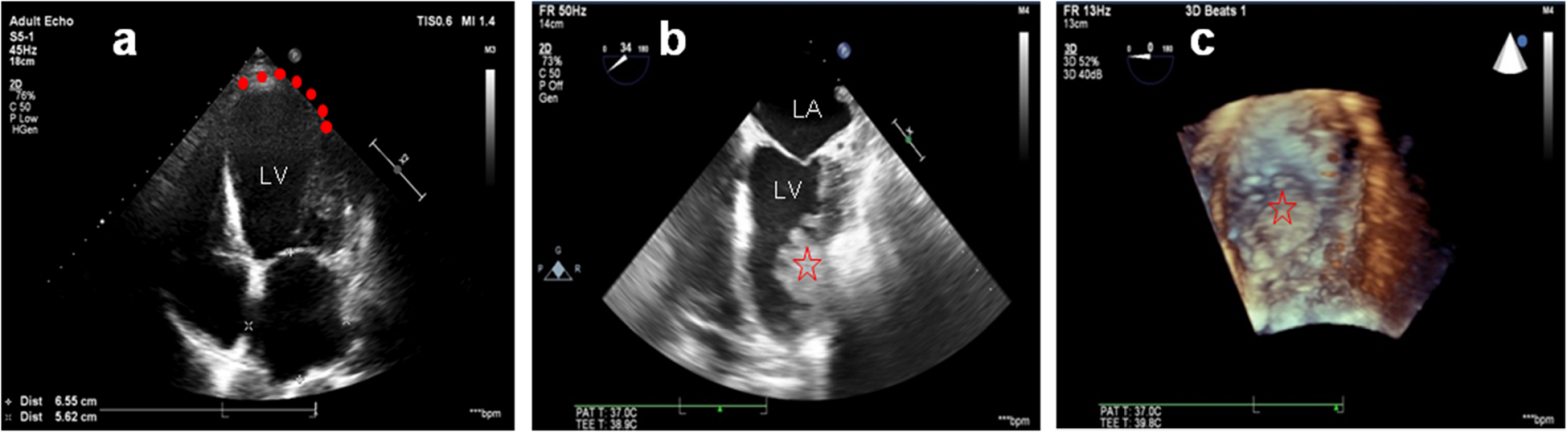
**a** TTE showing a large left ventricular aneurysm. Note the aneurismal dilation and the abnormal thin-wall (red dots) in the apex and anterior regions. **b** 2-dimensional TEE and **c** 3-dimensional TEE showing the left ventricular mural thrombus (red star). LA: Left atrium; LV: Left ventricle


A 67-year-old male has a history of old myocardial infarction, cardiac stents, apical LVA for almost 8 years and newly was diagnosed rectal cancer needing surgery. The patient was posted for non-cardiac surgery (Hartmann’s procedure) and stopped aspirin (100 mg QD) for 10 days to reduce the risk of bleeding. The preoperative cardiac troponin-l level stabilized at 0.07–0.078 ng/mL. Electrocardiogram (ECG) showed “persistent” Q-waves and elevated ST-segment with relevant inverted T waves in leads V2–V5, suggesting a remote healed anterior wall myocardial infarction (Fig. 1). Transthoracic echocardiogram (TTE) examination showed akinesia of the apical, ventricular septum and anterior wall of left ventricular with formation of an apical LVA, bulging 26 × 51 mm (Fig. 2a). Also, left ventricular dysfunction with ejection fraction of 46.7% and mild mitral regurgitation were found. But 2 weeks later seeing the TEE image on the stage, we were surprised to find that the result of TEE examination after induction of anesthesia was different from that of TTE preoperatively. Apart from the known extensive LVA, a well-circumscribed mural thrombus (30 × 30 mm) was visualized clearly (Fig. 2b, c and video). TEE is not a routine monitoring in non-heart surgery. And there were no suspicious symptoms occurred. This is because his left ventricular myocardial contractility was severely damaged with poor ejection fraction and we don’t see such patients very often. In order to decrease the risk of anesthesia for patients with chronic aneurysm, TEE was done to make real monitoring cardiac function intraoperatively. Fortunately, no thromboembolic or bleeding accidents happened intraoperatively. When the surgery was over, the serum troponin-l was 0.07 ng/mL and was not worsened comparing to the preoperative conditions. After surgery, in addition to continue taking antiplatelet therapy with aspirin, the patient was also treated with subcutaneous calcium nadroparin with frequency of twice a day (0.4 ml-4100 UI) for 5 days. The patient did well postoperatively, and 5 days later, TTE re-examination did not find LVMT. The patient discharged with good recovery on postoperative day 10. He was followed up for 2 years after the surgery, and there were no cardiovascular accidents occurred.

**Additional file 1.** Video of 3-dimensional TEE showing the left ventricular mural thrombus.

## Discussion and conclusions

Wall motion abnormalities, particularly LVA after myocardial infarction, lead to blood pooling and thrombus secondary formation, put the patient at greater risk for systemic embolization and cardioembolic stroke [4]. In this case described, the patient had long-standing cardiac stents and LVA nearly 8 years that had been optimized by antiplatelet therapy. LVMT was found on the day of surgery. The detection of LV thrombi is an incidental finding without definite age of thrombus. Diagnoses of thrombus age or classification in structural is not yet possible with existing technologies. So, the information about time and cause of thrombus formation should be discussed, because it is about what is right and wrong in what we did in preoperative preparation. There are two scenarios: either older thrombi not be found by less sensitive TTE, or fresh mural thrombosis due to aspirin withdraw.

First possibility, LVMT may have been in the left ventricular all along only because the preoperative TTE failed to identify its presence. As is known to all, echocardiography is one of the most widely diagnostic tests in current era because of lower cost, safer and without traumatic effect. In 2006, Srichai MB et al. [5] reported that small size thrombi (< 1 cm) is difficult to be detected by both TTE and TEE. Also, it has been reported that thrombi missed by TTE has small size and was located at apex (1 × 1 cm) and basal inferior aneurysm (1.3 × 3 cm) [6]. For our patient, the mural thrombus is big and protruding in shape (3 × 3 cm) and is not in nook. It is highly unlikely that the TTE failed to diagnose the presence of LVMT. In addition, he was examined with full-body PET-CT preoperatively, and there was no subclinical cerebral ischemia or embolization. The possibility that thrombus may have developed other times in the past was quite low.

The troponin-l was stabilized and no new myocardial infarction occurred. It is greatly possible that the mural thrombus is fresh which formed during the period of stopping aspirin. The usage of aspirin as antiplatelet and antithrombotic agent has been for several decades. However, stop or continuation aspirin before elective surgery has long been in debate. The surgeon analyzed the risk of hemorrhage before the open abdominal surgery for the patient and then ordered an aspirin treatment temporary stop for 10 days. Coexistence of wall motion abnormalities, preoperative aspirin discontinuation and without bridging with perioperative low-molecular-weight heparin, physical activity reduction and prolonged fasting are associated with an increased risk of blood hypercoagulability and thrombosis.

Current guidelines recommend that vitamin K antagonist is the first choice of treatment for cardiac thrombus [7]. There has been an increasing trend of studies using non-vitamin K antagonist oral anticoagulants (NOAC) for the treatment of LV thrombus [8, 9]. The researchers confirmed the safety and feasibility of using NOAC in patients with LV thrombus. In order to avoid the perioperative bleeding risk, we did not carry out the therapy of local anti-coagulation or thrombolysis, even though TEE detected LVMT. We encourage the patient to start early ambulation and resume aspirin therapy on the 2nd post-operative day to correct blood hypercoagulability. As TTE re-examination postoperative 5 days showed no evidence of LVMT, anticoagulative therapy was not applied. He was followed-up for 2 years and confirmed no systemic embolism happening.

Preoperative LAV/LVMT risk assessment is a vital step in reducing perioperative cardiac complications and mortality in patients undergoing non-cardiac surgery, which was operated by surgeons, cardiologists and anesthesiologists. We need to evaluate the pros and cons of preoperative withdrawal of aspirin in patients with cardiovascular disease undergoing non-cardiac surgery. Once patients stop taking antiplatelet agent, it might increase the risk of thrombus and should closely monitor the patient during this period. Furthermore, bridging with perioperative low-molecular-weight heparin is advisable.

## Limitations

The first TTE examination is 2 weeks before surgery and no TTE re-examination was applied during cessation of aspirin. We failed to detect thrombosis in time.

## Data Availability

All the data and information supporting the conclusion are presented in the article.

## Abbreviations

LVMT: Left ventricular mural thrombus
LVA: Left ventricular aneurysm
TEE: Transesophageal echocardiography
TTE: Transthoracic echocardiogram
ECG: Electrocardiogram
